# Sex as a Predictor of Dignity-Related Distress Among Patients with Advanced Cancer

**DOI:** 10.1089/pmr.2025.0012

**Published:** 2025-05-26

**Authors:** Hong Chen, Michiyo Mizuno, Tomoko Ito

**Affiliations:** ^1^ Graduate School of Comprehensive Human Sciences, University of Tsukuba, Tsukuba, Japan.; ^2^ Institute of Medicine, University of Tsukuba, Tsukuba, Ibaraki, Japan.

**Keywords:** advanced cancer, dignity-related distress, sex

## Abstract

**Objective:**

To explore the role of sex as a predictive factor for dignity-related distress among advanced cancer patients.

**Methods:**

The study employed a cross-sectional survey design, utilizing a Chinese version of the Patient Dignity Inventory (PDI). The participants were 294 patients with advanced cancer who were receiving treatment at a hospital in Xinjiang, China. Univariate analyses were conducted to compare scores on the total PDI and its five dimensions between sexes. Hierarchical multiple regression analyses were then performed with the PDI scores that exhibited significant sex differences designated as the dependent variable.

**Results:**

The total and existential distress subscale PDI scores were found to be significantly higher in men than in women. However, this sex disparity in the total PDI score was not statistically significant in the multivariable model. The multivariable model revealed that factors such as sex, the method of medical care payment, cancer stage, treatment status, and performance status were significantly associated with existential distress.

**Conclusions:**

The present findings suggest that the disparities in dignity-related distress between men and women can be better understood by examining how a patient’s sex affects the experience of existential distress. The findings also show that men tend to need more support to protect their dignity in terms of their sex roles than women. Also, addressing existential distress in men can play a crucial role not only in reducing dignity-related distress but also in addressing clinical issues such as performance status.

## Introduction

Dignity is a fundamental aspect of human life and an essential element of nursing practice. In 2002, Chochinov et al. published a dignity model for terminally ill patients.^1^ This model comprised three primary elements: illness-related concerns; a dignity-conserving repertoire, including personal actions; and a social dignity inventory of factors that affect a person’s sense of dignity.^1^ It is of utmost importance that the dignity of all patients be afforded equal respect. However, there are several reasons why the sense of dignity of terminally ill patients may be threatened, including anxiety around dying and feelings of helplessness and hopelessness. During the terminal stage of advanced cancer, patients often experience a range of distressing symptoms and conditions.^2^ Chochinov et al. subsequently developed the Patient Dignity Inventory (PDI) as a tool to measure dignity-related distress among terminally ill patients.^3^ The extent to which a patient experiences dignity-related distress and the underlying source of this distress are largely contingent upon a patient’s attributes and circumstances.

Sex is one of the attributes that can influence a patient’s experience of dignity-related distress.^4^ Note, “gender” is understood as a cultural and social construct that encompasses the roles, behaviors, and expectations imposed by society on individuals based on their perceived sex, distinguishing it from “sex,” which refers to the biological characteristics that define men and women.^5^ In the present study, we concentrated on the role of sex as a predictive factor for dignity-related distress in patients with advanced cancer. The effects of sex may be due to differences in biological, physical, and psychosocial aspects between men and women.^6,7^ For example, in Chinese culture, the traditional sex roles of men working outside the home and women performing household tasks remain strongly entrenched.^8^ The values attached to such sex roles may also affect the dignity of cancer patients. Therefore, it is important to take sex into account as a significant factor predicting dignity-related distress among advanced cancer patients so that effective, personalized care can be planned and implemented.

Sex shapes how individuals perceive and manage their illness, including dignity-related distress. A Japanese study reported that men were more inclined to delay seeking professional psychological support for distress.^9^ Other studies exploring sex differences in cancer patients have found greater anxiety among women than men,^7^ but a greater incidence of suicide among men than women, with higher rates reported in Asia.^10^ These complex results from previous studies suggest that the concept of self-dignity may be interpreted in different ways by men and women, influenced by their own experiences and, in turn, affecting their coping mechanisms. Consequently, patients with advanced cancer may face challenges that are strongly influenced by cultural views and expectations in terms of their sex, and dignity-related distress may manifest differently for men and women. To improve the quality of care for patients with advanced cancer, sex differences in dignity-related distress must be considered throughout the patient journey.

There is limited discussion in the existing literature about the role of sex in dignity-related distress among cancer patients. Some studies suggest that women may be more susceptible to dignity-related distress than men, as posited by Cao et al.,^11^ but the findings on the effects of sex have been inconsistent. For example, in a study from China, Wang et al. reported no statistically significant difference in dignity-related distress between men and women among cancer patients.^12^ Given these discrepancies, further investigations into the effects of sex differences on dignity-related distress are warranted. Furthermore, much of the existing literature on dignity-related distress in cancer patients has concentrated on urban or more developed regions of China, and there is limited information regarding dignity-related distress in cancer patients from less developed areas, such as Xinjiang Province in northwest China.

With medical advances and increased longevity, the number of patients with advanced cancer has increased in China. In 2022, there were approximately 4.82 million new cancer diagnoses in China, with 2.53 million cases among men and 2.29 million among women.^13^ Of note, the proportion of advanced cancer diagnoses is particularly high, with more than 52% of cancer cases being classified as advanced.^14^ In Xinjiang, in northwest China, men account for 51.66% of the population and the province has a men-to-women ratio of 106.85:100.^15^ Xinjiang has lower economic levels (ranking 21st of 31 provinces), and 7.42% fewer people aged ≥60 years than the national average.^15^ Xinjiang is notable for its cultural diversity; in particular, it is a Muslim enclave in China, with Muslims comprising 97.36% of the minority population and 57.96% of the total population.^16^ Given this unique demographic, health care, economic, and cultural landscape, it is likely that there are sex differences in dignity-related distress within this region. Therefore, the aim of the present study was to use the PDI to investigate sex as a predictive factor of dignity-related distress among patients with advanced cancer in Xinjiang, China.

## Methods

### Study setting

This study was a cross-sectional investigation of patients with advanced cancer in Xinjiang, China.

### Participants

Study participants were recruited from the Inpatient Oncology Department and Day Treatment Clinic of the Seventh Affiliated Hospital of Xinjiang Medical University between July 2023 and November 2023. The study included patients who had advanced cancer (Stages III or IV), were aged ≥18 years, had knowledge of their cancer diagnosis, and could communicate in Mandarin. Patients were excluded if they were assessed as having unstable or severe medical conditions, such as severe heart failure or severe forms of chronic obstructive pulmonary disease, had a history of cognitive impairment, or were unable to provide informed consent.

### Ethical approval

The study was conducted in accordance with the ethical standards set forth by the ethics review committees of the University of Tsukuba, Japan, with which the corresponding author is affiliated, and the Seventh Affiliated Hospital of Xinjiang Medical University, China, where the survey was conducted. Written informed consent was obtained from all patients prior to data collection.

### Indicators

Demographic data were collected for the study population, including age, sex, education status, religious affiliation, employment status, family monthly per-capita income, medical insurance, and sources of social support. In the present study, we report on sex differences between men and women participants, defined according to biological characteristics. For all participants, we recorded the type of cancer, cancer stage, time since diagnosis (in months), present treatment status, and performance status (Eastern Cooperative Oncology Group [ECOG] Performance Status scale).

The PDI was developed by Chochinov et al. in 2008 for terminally ill patients and was subsequently translated into Chinese by Ge et al. for palliative cancer patients.^3,17^ The PDI assesses various sources of dignity-related distress across five domains: symptom distress; existential distress; dependency; peace of mind; and social support.^2,3,18^ The revised Chinese version has demonstrated satisfactory reliability and validity, as evidenced by Cronbach’s alpha of 0.92 for total scores.^17^ The PDI assesses the severity of dignity-related distress in terminally ill patients and comprises 25 items across five dimensions: symptomatic distress (Items 3, 5–9); existential distress (Items 4, 11–14, 18); independence (Items 1, 2, 20); social support (Items 15–17); and peace of mind (Items 21, 22, 25).^17,18^ Items 10, 19, 23, and 24 on the PDI are not assigned to any of the five dimensions.^3^ Total scores on the PDI range from 25 to 125 points, and higher total scores indicate higher levels of perceived distress,^3,17^ which is classified into three categories: mild (25–50 points), moderate (51–75 points), and severe (76–125 points).^12^

### Statistical analysis

Patients characteristics were dichotomized (Table 1) and mean total PDI scores were compared between groups using the Mann-Whitney *U* test. To investigate sex differences, we compared total and subscale PDI scores between men and women using the Mann-Whitney *U* test.

**Table 1.** Total Patient Dignity Inventory Scores According to Patient Characteristics in the Entire Study Cohort (*N* = 294)

	Men (*n* = 158)*n* (%)	Women (*n* = 136)*n* (%)	Total (*N* = 294)*N* (%)	Total *PDI* score	*U*	*p* value
Age (years)						
<60	68 (43.0)	56 (41.2)	124 (42.2)	50.8 ± 12.2	9067.00	0.041
≥60	90 (57.0)	80 (58.8)	170 (57.8)	47.9 ± 10.7		
Education status						
<High school	73 (46.2)	63 (46.3)	136 (46.3)	50.6 ± 11.4	9261.00	0.041
≥High school	85 (53.8)	73 (53.7)	158 (53.7)	47.8 ± 11.3		
Religious affiliation						
Islam	35 (22.2)	33 (24.3)	68 (23.1)	49.0 ± 11.5	7545.00	0.821
Other^a^	123 (77.8)	103 (75.7)	226 (76.9)	49.3 ± 11.1		
Marital status						
Single	22 (13.9)	26 (19.1)	48 (16.3)	50.2 ± 11.7	5451.50	0.401
Married	136 (86.1)	110 (80.9)	246 (83.7)	48.9 ± 11.4		
Employment status						
Working	51 (32.3)	30 (22.1)	81 (27.6)	49.2 ± 12.0	8502.00	0.848
Not working^b^	107 (67.7)	106 (77.9)	213 (72.4)	49.1 ± 11.2		
Family monthly per-capita income (Yuan)						
<3000	120 (75.9)	109 (80.1)	150 (51.0)	50.1 ± 11.1	9511.00	0.077
≥3000	38 (24.1)	27 (19.9)	144 (49.0)	48.0 ± 11.6		
Payment method for medical care						
Multiple medical insurance^c^	21 (13.3)	22 (16.2)	43 (14.6)	43.8 ± 11.0	3670.50	0.001
Other^d^	137 (86.7)	114 (83.8)	251 (85.4)	50.0 ± 11.2		
Sources of social support						
Spouse	122 (77.2)	87 (64.0)	209 (71.1)	49.7 ± 10.9	7856.00	0.120
Nonspouse	36 (22.8)	49 (36.0)	85 (28.9)	47.6 ± 12.6		
Diagnosed with digestive system cancer						
Yes^e^	89 (56.3)	54 (39.7)	143 (48.6)	50.1 ± 11.8	9733.50	0.144
No^f^	69 (43.7)	82 (60.3)	151 (51.4)	48.2 ± 11.0		
Cancer stage						
III	62 (39.2)	66 (48.5)	128 (43.5)	46.8 ± 11.8	8289.00	0.001
IV	96 (60.8)	70 (51.5)	166 (56.5)	50.9 ± 10.8		
Time since diagnosis						
<6 months	58 (36.7)	40 (29.4)	98 (33.3)	48.3 ± 11.7	8982.00	0.365
≥6 months	100 (63.3)	96 (70.6)	196 (66.7)	49.5 ± 11.3		
Present treatment status						
With treatment^g^	153 (96.8)	122 (89.7)	275 (93.7)	49.7 ± 11.4	1512.50	0.002
Without treatment^h^	5 (3.2)	14 (10.3)	19 (6.5)	41.2 ± 9.3		
ECOG performance status^i^						
Level 0	37 (23.4)	32 (23.5)	69 (23.5)	39.5 ± 8.7	2785.50	<0.001
Levels 1–4	121 (76.6)	104 (76.5)	225 (76.5)	52.0 ± 10.5		

Unless indicated otherwise, data are given as the mean ±SD or *n* (%). Significance was evaluated using Mann-Whitney U tests.

^a^ Other includes no religion (*n* = 75), Confucianism (*n* = 145), Buddhism (*n* = 2), and Christianity (*n* = 4).

^b^ Includes retirees (*n* = 167), students (*n* = 4), and housewives (*n* = 42).

^c^ Resident medical insurance + commercial insurance/critical illness insurance (*n* = 43).

^d^ Includes resident medical insurance (*n* = 249) and self-funded payment (*n* = 2).

^e^ Digestive system cancers include colorectal (*n* = 56), gastric (*n* = 32), liver (*n* = 26), esophageal (*n* = 18), and pancreatic (*n* = 11) cancers.

^f^ Nondigestive system cancers include lung (*n* = 76), breast (*n* = 30), cervical (*n* = 16), ovarian (*n* = 10), kidney (*n* = 7), prostate (*n* = 5), bladder (*n* = 3), nasopharyngeal (*n* = 1), thyroid (*n* = 1), and laryngeal (*n* = 1) cancers.

^g^ Treatments include chemotherapy (*n* = 168), chemotherapy and other combination therapies (*n* = 70), targeted therapy (*n* = 21), and immunotherapy (*n* = 16).

^h^ “Without treatment” refers to patients who had undergone conservative treatment.

^i^ Performance status was assessed using the Eastern Cooperative Oncology Group (ECOG) Levels 0–5 scale to determine a patient’s functional status in oncology. Level 1, *n* = 172; Level 2, *n* = 31; Level 3 or 4, *n* = 22.

PDI, Patient Dignity Inventory.

In this study, we used hierarchical multiple regression analysis with the total PDI score as the dependent variable. In Model 1, only sex was included as the independent variable to control for its influence on total PDI scores. Model 2 included age, employment status, payment method for medical care, cancer stage, present treatment status, and ECOG performance status. Similarly, we performed multivariable regression analysis using the PDI existential distress score, for which significant sex differences were observed, as a dependent variable in Models 1 and 2.

Continuous variables are presented as the mean ±SD, and categorical data are presented as numbers and percentages. Data were analyzed using SPSS 28.0. All statistical tests were two-tailed and significance was set at *p* < 0.05.

## Results

Initially, 322 patients were enrolled in the study. Of these, 28 (8.7%) were excluded: 24 (7.5%) because they could not communicate in Mandarin and 4 (1.2%) because they declined to participate. Thus, 294 (91.3%) patients were included in the study and completed the questionnaire (158 [53.7%] men and 136 [46.3%] women).

The revised Chinese version of the PDI was used in the present study to assess dignity-related distress in terminally ill patients. The PDI exhibited satisfactory reliability and validity, with Cronbach’s alpha of 0.90 for the total PDI score, and values of 0.83, 0.72, 0.92, 0.78, and 0.71 for the symptomatic distress, existential distress, independence, social support, and peace of mind dimensions, respectively.

### Total PDI scores according to patient characteristics

Table 1 presents mean total PDI scores according to dichotomized patient characteristics for the entire study cohort. Mean total PDI scores differed significantly with age (U = 9067.00, *p* = 0.041), employment status (U = 9261.00, *p* = 0.041), payment method for medical care (U = 3670.50, *p* = 0.001), cancer stage (U = 8289.00, *p* = 0.001), present treatment status (U = 1512.50, *p* = 0.002), and ECOG performance status (U = 2785.50, *p* < 0.001).

### PDI total and subscale scores according to sex

The mean total PDI scores for the entire study cohort (49.1 ± 11.4) indicated a mild level of dignity-related distress (Table 2). Mean total PDI scores indicated a moderate level of dignity-related distress for men (total score 50.5 ± 11.4) and a mild level of dignity-related distress for women (total score 47.5 ± 11.2). The distribution of subscale scores for the symptom distress and existential distress dimensions was similar equivalent to that of the total PDI score (data not shown). The distribution of scores for the dependency and social support dimensions were positively and significantly skew (skewness: 1.75 and 1.47, respectively; kurtosis: 2.95 and 3.18, respectively). Similarly, the distribution of peace of mind scores was positively skewed (skewness: 0.71; kurtosis: 0.12), although to a lesser extent than scores for the dependence and social support dimensions.

**Table 2.** Comparison of Total and Subscale Scores on the Patient Dignity Inventory in the Entire Cohort and According to Sex

	Score range	Men (*n* = 158)	Women (*n* = 136)	Total (*N* = 294)	*U*	*p* value
Total PDI score	25–125	50.5 ± 11.4	47.5 ± 11.2	49.1 ± 11.4	9160.00	0.029
PDI subscales						
Symptom distress	6–30	14.3 ± 4.2	13.8 ± 4.3	14.1 ± 4.3	9756.50	0.173
Existential distress	6–30	13.8 ± 3.1	12.7 ± 3.3	13.3 ± 3.2	8567.00	0.003
Dependency	3–15	5.0 ± 2.8	4.6 ± 2.5	4.8. ±2.7	9884.00	0.193
Peace of mind	3–15	5.5 ± 1.9	5.3 ± 1.8	5.4 ± 1.9	9862.00	0.218
Social support	3–15	3.9 ± 1.2	3.8 ± 1.1	3.9 ± 1.1	10,112.00	0.339

Unless indicated otherwise, data are presented as the mean ±SD. Significance was evaluated using Mann-Whitney *U* tests.

PDI, Patient Dignity Inventory.

Significant differences were observed between men and women in mean total PDI scores (U = 916.00, *p =* 0.020) and existential distress scores (U = 8567.00, *p* = 0.003; Table 2). The total PDI scores exhibited a positive skew, with fewer higher scores than lower scores (skewness: 0.10; kurtosis: –0.97; median: 48.0).

### Hierarchical regression analysis of total and existential distress subscale scores on the PDI

The findings of the hierarchical multiple regression analysis with total PDI score as the dependent variable are presented in Table 3, while the results of the hierarchical multiple regression analysis with existential distress dimension score as the dependent variable are shown in Table 4. In the hierarchical multiple regression analysis with total PDI scores as the dependent variable, Model 1 was found to be significant (*R*^2^ = 0.02, F = 5.21, *p* = 0.023). The addition of covariates in Model 2 resulted in an improved fit (*R*^2^ = 0.33, F = 20.16, *p* < 0.001), accompanied by a significant increase in the *R*^2^ value from 0.02 to 0.33 (Δ*R*^2^ = 0.31; *p* < 0.001). The effects of age, payment method for medical care, cancer stage, present treatment status, and ECOG performance status on total PDI scores were statistically significant (*p* < 0.05).

**Table 3.** Hierarchical Regression Results for Total Scores on the Patient Dignity Inventory

Variable	B	95% CI for B		SE B	β	*R* ^2^	Δ*R*^2^
		LL	UL				
Model 1						0.02	0.01^*^
Constant	0.01	0.00	0.03	0.01			
Sex	–0.03^*^	–0.05	0.00	0.01	–0.13		
Model 2						0.33	0.31^***^
Constant	–0.16^***^	–0.21	–0.11	0.03			
Sex	–0.02	–0.04	0.00	0.01	–0.09		
Age	–0.02^*^	–0.04	0.00	0.01	–0.10		
Education status	–0.02	–0.04	0.01	0.01	–0.07		
Payment method for medical care	0.05^**^	0.02	0.08	0.01	0.17		
Cancer stage	0.03^**^	0.01	0.05	0.01	0.15		
Present treatment status	0.05^*^	0.01	0.09	0.02	0.11		
ECOG performance status	0.11^***^	0.09	0.13	0.01	0.45		

^*^ *p* < 0.05.

^**^ *p* < 0.01.

^***^ *p* < 0.001.

Model 1: Control variable = sex; dependent variable = log_10_ (total PDI score).

Model 2: Control variable = sex; covariates = age, education status, payment method for medical care, cancer stage, present treatment status, Eastern Cooperative Oncology Group (ECOG) performance status; dependent variable = log_10_ (total PDI score).

Dummy variables: (1) sex: men = 0, women = 1; (2) age: <60 years = 0, ≥60 years = 1; (3) education status: <high school = 0, ≥high school = 1; (4) payment method for medical care: multiple medical insurance = 0, other = 1; (5) cancer stage: Stage III = 0, Stage IV = 1; (6) present treatment status: yes = 0, no = 1; (7) performance status: ECOG Level 0 = 0, Levels 1–4 = 1.

CI, confidence interval; LL, lower limit; UL, upper limit.

**Table 4.** Hierarchical Regression Results for the Existential Distress Dimension on the Patient Dignity Inventory

Variables	B	95% CI for B		SE B	β	*R* ^2^	Δ*R*^2^
		LL	UL				
Model 1						0.03	0.02^*^
Constant	0.02	0.00	0.03	0.01			
Sex	–0.04^**^	–0.06	–0.01	0.01	–0.17		
Model 2						0.22	0.20^***^
Constant	–0.13^***^	–0.20	–0.07	0.03			
Sex	–0.03^*^	–0.05	–0.01	0.01	–0.13		
Age	–0.02	–0.04	0.01	0.01	–0.08		
Education status	–0.02	–0.04	0.01	0.01	–0.07		
Payment method for medical care	0.04^*^	0.00	0.07	0.02	0.12		
Cancer stage	0.04^**^	0.01	0.06	0.01	0.16		
Present treatment status	0.06^*^	0.01	0.10	0.02	0.12		
ECOG performance status	0.08^***^	0.05	0.11	0.01	0.31		

^*^ *p* < 0.05.

^**^ *p* < 0.01.

^***^ *p* < 0.001.

Model 1: Control variable = sex; dependent variable = log_10_ (existential distress score).

Model 2: Control variable = sex; covariates = age, education status, payment method for medical care, cancer stage, present treatment status, Eastern Cooperative Oncology Group (ECOG) performance status; dependent variable = log_10_ (existential distress score).

Dummy variables: (1) sex: men = 0, women = 1; (2) age: <60 years = 0, ≥60 years = 1; (3) education status: <high school = 0, ≥high school = 1; (4) payment method for medical care: multiple medical insurance = 0, other = 1; (5) cancer stage: Stage III = 0, Stage IV = 1; (6) present treatment status: yes = 0, no = 1; (7) performance status: ECOG Level 0 = 0, Levels 1–4 = 1.

CI, confidence interval; LL, lower limit; UL, upper limit.

In the hierarchical multiple regression analysis with existential distress subscale scores as the dependent variable, Model 1 was found to be significant (*R*^2^ = 0.03, *F* = 8.31, *p* = 0.004). The addition of covariates in Model 2 resulted in an improved fit (*R*^2^ = 0.22, *F* = 11.17, *p* < 0.001), with increase in the *R*^2^ value from 0.03 to 0.22 (Δ*R*^2^ = 0.19; *p* < 0.001). Even after adjusting for age, employment status, payment method for medical care, cancer stage, present treatment status, and ECOG performance status, sex remained a significant predictor in Model 2.

## Discussion

The findings of this study indicate that the study participants experienced mild dignity-related distress. This contrasts with other studies examining dignity-related distress in patients with advanced cancer that have reported moderate distress among patients, including a study of 150 Chinese patients (mean total PDI score 56.9)^11^ and a German study (mean total PDI score 51.6).^19^ In the present study, the distribution of scores for three of the five dimensions, namely dependency, social support, and peace of mind, exhibited a significant positive skew. This skewing suggests that a limited number of participants experienced severe dignity-related distress as indicated by these subscales, which could be one reason for the low total PDI scores in our study. Research supports this, with one study reporting that most cancer patients report mild (71%) or moderate (18%) distress, and only 5% of patients report severe distress.^12^

In our study sample, 23.1% of patients were Muslims, and there was no significant difference in the severity of dignity-related distress between Muslim and nonMuslim participants (including those with no religious affiliation). However, differences were observed in the severity of dignity-related distress according to dichotomized demographic and environmental factors (sex, age, employment status, and payment method for medical care), as well as clinical factors (cancer stage, present treatment status, and ECOG performance status).

In examining the effect of sex on dignity-related distress, which was the primary focus of this study, the level of distress among women was classified as mild, whereas that among men was classified as moderate. A previous study reported moderate distress levels overall among patients with advanced cancer, with mean total PDI scores of 54.0 ± 16.3 and 59.7 ± 16.2 in men and women, respectively.^11^ Comparable patterns were seen in scores for the two distress subscales, namely, symptom distress and existential distress. However, in the present study, a statistically significant difference between men and women was only observed for the existential distress subscale score. The PDI existential distress domain encompasses feelings and issues patients may grapple with as death approaches, illustrating concerns that include a lost sense of identity, feelings of worthlessness, changes in appearance, an inability to fulfill important roles, a perceived lack of meaning in life, and the feeling of being a burden to others.^3^ We confirmed the relationship between sex and existential distress on the PDI through multiple regression analysis, which revealed that the relationship remained significant even after adjusting for covariates other than sex. The results indicated that sex was a significant predictor of existential distress on the PDI, along with the method of payment for medical care and several clinical factors, including cancer stage, present treatment status, and ECOG performance status. However, although these clinical factors were able to predict overall dignity-related distress, sex did not emerge as a significant predictor. In contrast, age, rather than sex, was identified as a significant factor affecting overall dignity-related distress.

Previous studies have demonstrated that declines in physical health and function contribute significantly to an increase in dignity-related distress among cancer patients.^4,20^ The present study also showed that advanced cancer stage, undergoing medical treatment, and having a low ECOG performance status increased the likelihood of distress on the PDI, not only in terms of existential distress but also in other ways. ECOG performance status, which is an indicator of physical function and the ability to engage in daily activities, had a pronounced effect on dignity-related distress. Furthermore, even when considering the effects of these medical factors, men sex continued to predict a high level of existential distress related to dignity.

This study has revealed the significance of focusing on existential distress as a source of dignity-related distress for patients with advanced cancer at a hospital in the Xinjiang Uyghur Autonomous Region. Although the mean total PDI score for participants indicated mild distress, the finding that existential distress was more likely to cause dignity-related distress in men than in women highlights the need for careful assessment of the extent of existential distress, along with clinical indicators. In evaluating the source of dignity-related distress, it is essential to consider the cultural norms of China, including sex roles and the traditional roles of men and women within families, in addition to common psychosocial and environmental factors. Xinjiang’s unique cultural fabric, shaped by Confucian and Islamic values, emphasizes complementary sex roles: men as providers and women as nurturers. Addressing the interplay of cultural diversity, sex roles, and existential distress in patients with advanced cancer could enhance palliative care practices by fostering more foster more culturally sensitive and effective support systems.

### Limitations and future directions

The present study has several limitations that should be acknowledged. First, the sample was drawn from a single hospital, which may limit the generalizability of the findings to the broader population of patients with advanced cancer in China. Approximately one-third of the patients seen at the study site come from ethnic minorities, such as the Uyghurs and Kazakhs. In addition, approximately 25.0–37.7% of the health care staff at the hospital are from these ethnic minorities, helping to address communication issues with patients. Future research should aim to include a more diverse sample across multiple health care settings to enhance the representativeness of the findings. Second, this study used a cross-sectional design, which restricts the ability to establish causal relationships. Longitudinal studies would provide valuable insights into how dignity-related distress evolves over time in response to various factors, including treatment progress and changes in personal circumstances. In addition, further exploration of the interaction between sex, ECOG performance status, and cancer stage is warranted. Understanding how these variables collectively shape the experience of existential distress as identified on the PDI could inform the development of targeted psychological interventions. Finally, we cannot rule out effects of unobserved confounders.

## Conclusions

The mean total PDI score of participants in this study equated to mild distress. Men had significantly higher total PDI scores than women, thereby suggesting greater dignity-related distress. However, this sex difference was no longer evident in the multivariable model. Men also had significantly higher levels of existential distress on the PDI than women, even after adjusting the model for covariates. These findings underscore the need to develop tailored interventions to assess and address the unique needs of men and women cancer patients. Furthermore, the findings emphasize the importance of conducting further research across diverse populations to enhance clinical practice and improve patient care, particularly by incorporating existential distress assessments and sex-sensitive approaches in palliative care.

## Data Availability

Data are available on request due to privacy/ethical restrictions. The data that support the findings of this study are available on request from the corresponding author, M.M. These data are not publicly available, as they contain information that may compromise the privacy of research participants.

## Abbreviations Used

PDI: The Patient Dignity Inventory
ECOG Performance Status: Eastern Cooperative Oncology Group Performance Status
